# Supplementary material for: Learning curve and surgical outcome of robotic assisted colorectal surgery with ERAS program
Source: Sci Rep. 2022 Nov 29;12:20566. doi: 10.1038/s41598-022-24665-w (PMC9709162; doi:10.1038/s41598-022-24665-w)
Supplement: Supplementary file 1 — Supplementary Information 1. [file 41598_2022_24665_MOESM1_ESM.zip › LSC Raw data-Ñ[▒K/058/P_ñw╝╨░O▒Kñσ.pdf]

[redacted]

Pathologic diagnosis:
1. Rectum, laparoscopic low anterior resection --- Tubulovillous adenoma with focal high grade dysplasia. The bilateral cut ends and perirectal lymph nodes (0/25) are free of lesion.
2. Rectum, cut end, distal, resection --- Chronic inflammation.

Prognostic and predictive factor:
1. Degree of dysplasia: High grade.
2. Margin of stalk: Free.

Gross description:
The specimen consists of 1) a segment of colon, 11.5 cm in length and 6 cm in proximal cut end circumference, 8.5 cm in distal circumference, and perirectal soft tissue. A gray tan polypoid tumor, 3.8x2.7 cm, is noted, 2.5 cm to the nearest cut end. On cross section, no definite stromal invasion is idnetified. The both cut ends and radial margin are free of tumor grossly. 2) distal cut end, a piece of tan mucosa-covered soft tissue, 2.1x1.7x0.7 cm.
Representative part for section: A) proximal cut end B) distal cut end C) colon D-F) tumor G) specimen 2 H-K) lymph node.
#T-59300_2 #M-82630_2 0 1105 000000
#T-C4520_2 #M-09183_2 0 1105 000000

已電子簽章

敬告：本單僅供醫師參考使用，不具任何申請效力！！
請病患妥為保管，若不慎遺失需自負個人資料外洩責任！
